# Ecology and epidemiology of multidrug resistance *Salmonella* in fruit Bats and shared water sources in Bangladesh

**DOI:** 10.1371/journal.pone.0361701

**Published:** 2026-10-30

**Authors:** Md. Helal Uddin, Pronesh Dutta, Monjurul Islam, Md. Kaisar Rahman, Shariful Islam, Md. Rezaul Karim, Farhana Rahman, Jade K. Forwood, Sharmin Chowdhury, Jonathan H. Epstein, Mohammed Abdus Samad, Ariful Islam

**Affiliations:** 1 Faculty of Veterinary Medicine, Chattogram Veterinary and Animal Sciences University, Chattogram, Bangladesh; 2 Zoonotic Disease Research Program, Institute of Epidemiology, Disease Control and Research (IEDCR), Dhaka, Bangladesh; 3 Biosecurity Research Program, Gulbali Research Institute, Charles Sturt University, Wagga Wagga, New South Wales, Australia; 4 Faculty of Veterinary Medicine, Universiti Malaysia Kelantan, Kota Bharu, Kelantan, Malaysia; 5 Antimicrobial Resistance Action Center (ARAC), Animal Health Research Division, Bangladesh Livestock Research Institute, Savar, Bangladesh; 6 Bangladesh Forest Department, Agargaon, Sher-e-Bangla Nagar, Dhaka, Bangladesh; 7 One Health Science, Mt Kisco, New York, United States of America; Universidad San Francisco de Quito, ECUADOR

## Abstract

Antimicrobial resistance (AMR) is a growing global threat to both animal and human health, underscoring the importance of AMR surveillance across all sectors of the One Health triad, including wildlife. Fruit bats in Bangladesh frequently inhabit areas in close proximity to human settlements and livestock facilities, creating ecological conditions that facilitate the co-occurrence of AMR bacteria through direct contact or shared contaminated environments. A cross-sectional study was conducted to determine the prevalence of *Salmonella* spp. isolation and the proportion of antimicrobial-resistant isolates among confirmed positives, characterize resistance patterns across multiple antimicrobial classes, and identify associated risk factors in fruit bat populations and environmental water sources in Bangladesh. We collected 369 fecal samples from the roosts of two bat species, *Pteropus medius* and *Rousettus leschenaultii*, along with 22 water samples from water bodies utilized by humans, livestock, and bats. *Salmonella* spp. isolates were identified using selective agar culture, followed by biochemical testing and confirmation via polymerase chain reaction (PCR). Confirmed isolates underwent antimicrobial susceptibility testing via the Kirby-Bauer disc diffusion method against 12 antimicrobials. The prevalence of AMR *Salmonella* spp. was 7.9% (95% CI: 5.3–11.1%) in bat’s roost fecal samples and 31.8% (95% CI: 13.9–54.9%) in environmental water samples. Bat-derived isolates showed high resistance to tetracycline (93%), nalidixic acid (86%), sulfamethoxazole-trimethoprim (80%), and azithromycin (76%). Among the identified AMR isolates, 28 (96.5%) met the criteria for multidrug resistance (MDR), defined as resistance to three or more antimicrobial classes. Water-derived isolates also demonstrated high resistance, particularly to tetracycline (86%), amoxicillin-clavulanic acid (75%), azithromycin (73%), ceftriaxone (72%), and ciprofloxacin (71%). Multivariable logistic regression analysis revealed that the odds ratio (OR) of detection of MDR *Salmonella* spp. in fruit bats was 5.1 times higher during the summer season compared to winter. These findings highlight the evidence of MDR *Salmonella* spp. in fruit bats and shared environmental water sources in Bangladesh. The co-occurrence of resistant isolates across wildlife and environmental samples highlights the need for integrated AMR surveillance encompassing wildlife reservoirs and shared ecological interfaces. Strengthened antimicrobial stewardship, improved wastewater management, and coordinated One Health surveillance strategies are essential to mitigate the risk of zoonotic spillover and reduce further ecological overlap of MDR bacteria at the wildlife–livestock–human interface in Bangladesh and the broader South Asian region.

## Introduction

Antimicrobial resistance (AMR) is a major global challenge driven by its growing impact on population health, healthcare systems, and the global economy [1,2]. The overall incidence of diseases caused by resistant microorganisms will result in 10 million human deaths annually by 2050, surpassing many other leading causes of mortality [3,4]. While antibiotics have revolutionized the treatment of infectious diseases since the 20th century, their widespread misuse in human and animal health has fueled resistance in pathogenic bacteria and the spread of AMR across ecosystems [5,6]. Although much of the attention has been directed toward clinical settings, there is increasing recognition that the environmental dimension, including wildlife and ecosystem health, is critical in understanding the full scope of AMR [7,8], and that AMR determinants can also persist and evolve in environmental reservoirs, such as soil, water, and wildlife [9].

Wildlife, especially mammals, have emerged as important yet underappreciated reservoirs of antimicrobial-resistant organisms [10]. Despite limited exposure to antibiotics, wild animals acquire resistant bacteria through environmental contamination [11]. This contamination often stems from anthropogenic activities, including the discharge of hospital effluents containing resistant bacteria and unmetabolized antibiotics, pharmaceutical manufacturing waste, feces from treated livestock, and untreated municipal wastewater [12,13].

Among wild mammals, bats are the second-largest mammalian group, with over 1,300 species in the order Chiroptera [14,15]. Bats are the most abundant, diverse, and geographically dispersed vertebrates and are natural reservoirs of several pathogenic agents, many of which cause severe diseases and outbreaks in humans and livestock worldwide, such as the Nipah virus and Coronaviruses [16,17]. Though bats play a significant role in recent pandemics and disease spillover, the presence of AMR bacteria in bats has a crucial impact on global health [18], as they are an important reservoir and potential vector of AMR enteric bacteria [19].

*Salmonella* spp*.* are globally distributed and can be found in all warm-blooded animals and in the surrounding environment. Transmission of *Salmonella* spp. infection typically occurs through the ingestion of contaminated food or water with feces from infected hosts, posing both economic burdens on the livestock industry and serious public health concerns [20]. *Salmonella* spp. are among the most significant foodborne pathogens globally and the third largest cause of human mortality from diarrheal illnesses [21]. It is estimated that 90 million cases of gastroenteritis due to *Salmonella* spp. are reported globally, with 155,000 deaths annually [22]. Livestock and wild birds are considered significant sources of *Salmonella* spp. serotypes specially the non-typhoid salmonellae (NTS) that are pathogenic to humans [21]. Human encroachment on wildlife habitats leads to direct interactions between humans and wildlife species, such as hunting, as well as indirect interactions with other species, specially livestock living in close proximity, which influences the spillover of zoonotic bacterial infections to humans and other animals [23]. However, wild animals are also colonized by *Salmonella* spp. and harbor them in the gastrointestinal tract without showing any symptoms [24]. *Salmonella* spp. have been isolated from bats of different species in other countries [25–27]. Fruit bats live close to humans and livestock farms and share the same habitat and food resources [28,29], which may lead to bacterial infections in bats by taking fruits and water that are contaminated with the waste of antibiotic-treated humans and livestock [25]. There is increasing evidence of the spread of pathogenic, drug-resistant bacteria in wildlife [10], including bats, which are not treated with antimicrobial agents in free-ranging habitats [30]. Several studies have identified multidrug-resistant (MDR) *Salmonella* spp. in bats in several countries [26,31].

Bangladesh is home to three species of frugivorous bats that are abundant and known to roost and forage near human settlements: *Pteropus medius* (formerly *Pteropus giganteus*)*, Cynopterus sphinx*, and *Rousettus leschenaultii* [28]. Among the fruit bats. *P. medius* is the natural reservoir of Nipah virus (NiV) and is responsible for NiV transmission, with a < 70% case fatality rate with NiV in humans in Bangladesh through consumption of date palm sap contaminated with bat saliva and excreta [32], resulting in a lower concentration of research on zoonotic bacterial transmission in bats in Bangladesh.

*Salmonella* Typhi infection is common in Bangladesh, resulting in typhoid fever and gastroenteritis in humans [33]. Besides, *Salmonella* spp. has been identified in poultry [34] and animal products [35], indicating the source of zoonotic transmission to humans. It has been reported that human infection with *Salmonella* spp. is associated with eating papaya [36], a common fruit consumed by birds and fruit bats in Bangladesh [37]. However, bats can get the infection of zoonotic *Salmonella* spp. from humans and animals as they share common water sources for drinking [37], where people and livestock use the same water source for bathing, and these water sources are often contaminated with sewage [38]. Developing countries such as Bangladesh are vulnerable to AMR due to their limited surveillance and weak healthcare facilities [39]. AMR *Salmonella* spp. have been identified in food products and by-products from livestock and poultry in Bangladesh [40], posing a significant public health challenge. While most research on AMR bacteria in Bangladesh has focused on humans, livestock, and poultry, few studies have identified bacterial infections in fruit bats in Bangladesh and provided antibiograms [37]. Bats often forage and roost near human settlements, making it crucial to understand the eco-epidemiology of AMR at the bat-human-livestock interface. Hence, this study aimed to determine the prevalence of *Salmonella* spp. isolation and the proportion of antimicrobial resistance among confirmed isolates, characterize resistance patterns across multiple antimicrobial classes, and identify ecological and environmental factors associated with AMR in *Salmonella* spp. detection in free-ranging fruit bats and shared water sources at rural and urban human–wildlife–livestock interfaces in Bangladesh.

## Materials and methods

### Ethical approval

The study protocol was reviewed and approved by the Animal Experimentation Ethics Committee at the Chattogram Veterinary and Animal Sciences University (CVASU/Dir (R&E) AEEC/2015/751), dated 05/09/2016. The committee approved the methodology, which included a study design based on non-invasive sampling of bat fecal and water sources, thereby avoiding the capture, handling, or disturbance of bats. All sampling procedures adhered to institutional animal ethics guidelines and established wildlife welfare standards. Fieldwork was conducted under the supervision of experienced researchers, and personnel safety protocols were strictly observed throughout the study. Additionally, bat sampling was carried out under permission granted by the Bangladesh Forest Department (Permit Letter No.: 22.01.0000.101.23.2016.2418, dated 23 June 2016).

### Study design and duration

A cross-sectional study was conducted between December 2016 and June 2017 across four districts (Dhaka, Faridpur, Rajbari, and Madaripur) within the Dhaka division of Bangladesh. A total of eleven bat roosts were identified across the four study districts, based on the presence of established bat habitats and documented human-bat interactions, including five in Dhaka, four in Faridpur, and one each in Madaripur and Rajbari. In addition, 22 water bodies within a 5 km radius of the identified bat roosts were surveyed to assess whether humans, livestock, domestic animals, wildlife, and bats shared and used these common water sources (Fig 1). We visited each water body early in the morning (5:00 AM to 6:00 AM) and during dusk (5:30 PM to 6:30 PM) to observe the bat drinking behavior and human and animal use of these water bodies during the morning (5:00 AM to 11:00 AM) and afternoon (12:00 PM to 5:30 PM), and collected the water samples. Observed human activities included drinking, bathing, fishing, swimming, and washing utensils and clothes, while livestock and wildlife were also frequently observed using the same water sources.

**Fig 1:**
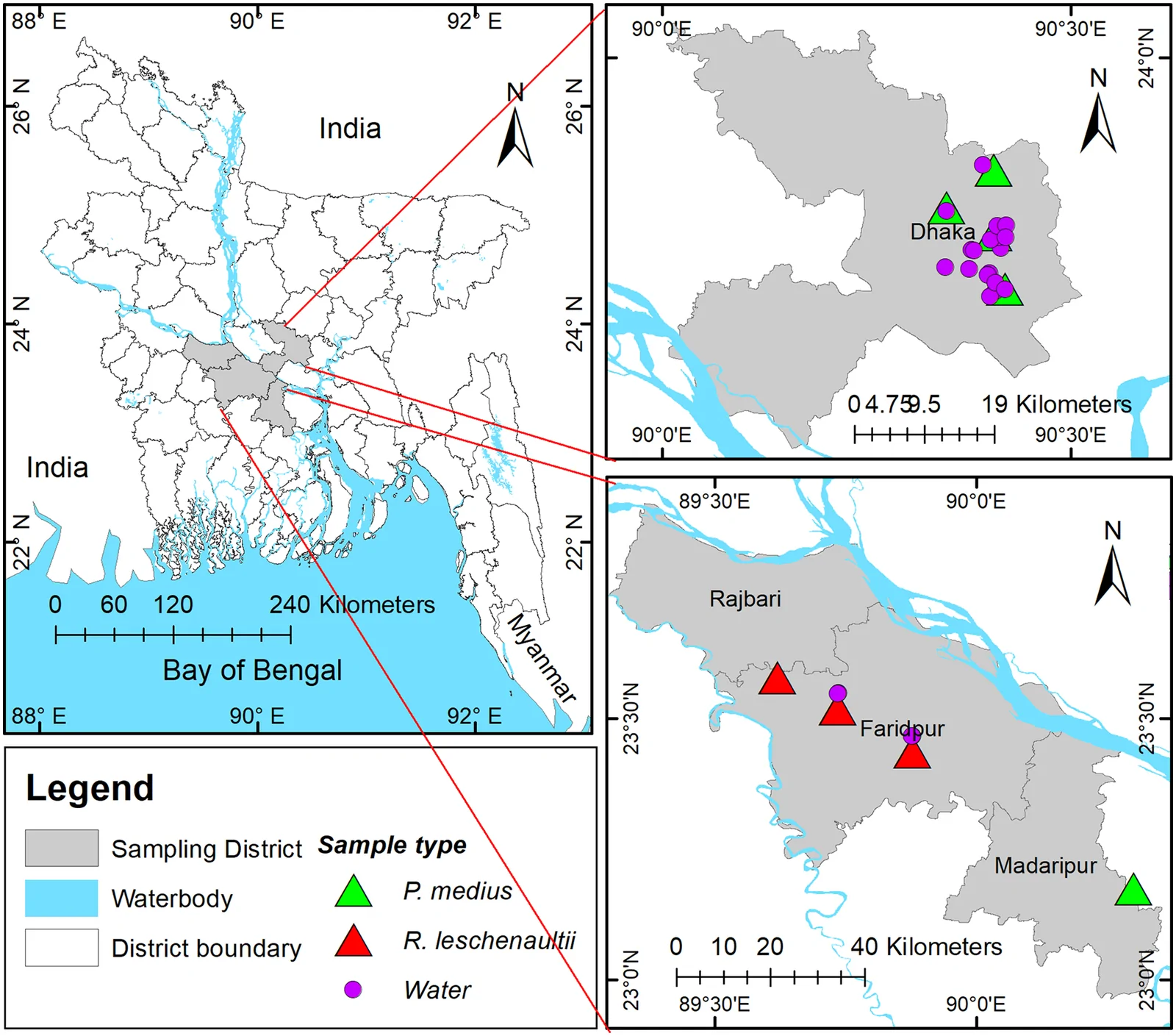
Geographical locations of *P. medius* and *R. leschenaultii* bats’ roosts and water bodies in the study sites. Bat roosts were identified as triangular (▲) symbols, and circular (O) symbols referred to the locations of water bodies. The map was created using a shapefile from the freely accessible GADM database (GADM: www.gadm.org) [41].

### Sample size calculations

The prevalence of antimicrobial-resistant microbes in wildlife is poorly understood. There is no previous record of AMR prevalence in bats in Bangladesh. So, the maximum sample size was calculated based on assuming a 50% prevalence (p = 0.5) as per the available literature [42], taking 5% precision (d = 0.05) and 95% confidence interval (i.e., Z = 1.96), following the simple random sampling formula [43]


$$\begin{array}{c} n=z^{\text{2}}\times \;\frac{p(\text{1}-p)}{d^{\text{2}}} \end{array}$$

Where n = desired sample size, Z = the standard normal deviation, usually set at 1.96 at 95% confidence level, p = expected prevalence = 0.5, and d = precision (5%, so d = 0.05). Sampling and sample collection were done upon judgment and convenience sampling as described for wildlife by Morrison et al. [44]. According to the formula we needed 385 bats samples, but we were able to collect a total of 369 fecal samples from the bat roosts of two available and widely distributed bat species (*P. medius,* and *R. leschenaultii*) in the study area, during the field survey of the following research area.

### Sample collection, processing, and storage

Polythene sheets (five per roost tree; 6 feet × 4 feet each) were placed beneath and around the base of each roost tree between 2:00 am and 4:00 am, depending on roost size and available ground space, following the methodology described by Islam et al. [45]. Fecal sample collection was conducted between 05:00 and 06:00 AM, coinciding with the return of the majority of bats to the roost. Freshly voided fecal samples were collected using a non-invasive approach, as outlined by Swift et al. [46], in accordance with established ethical and animal welfare standards. We collected a single non-invasive fecal swab from an intact fecal deposit large enough to be swabbed without contamination from adjacent droppings. Only fresh, intact single, well-formed fecal pellets were selected for each swab, avoiding pooled or smeared material from multiple deposits. Each sterile swab was gently rotated over the surface of the selected fecal pellet to collect sufficient material while avoiding overload. To minimize the likelihood of repeatedly sampling droppings from the same individual bat, we took only four distinct spatially separated, well-formed fecal pellets, one from each corner of the polyethylene collection sheet, and noted the spatial separation between sampled droppings. After sampling, each collection location was marked to prevent duplicate collection [47]. In addition, the spatial distance between sampled pellets was recorded as an additional measure to reduce the likelihood of repeated sampling from the same individual bat. Although individual bats were not captured or identified, this non-invasive sampling protocol substantially reduced the risk of pseudoreplication under field conditions. Bat roost fecal samples were collected during two seasonal periods: winter (December to March) and summer (April to June). After collection, samples were individually transferred into Falcon tubes containing 10 mL of Buffered Peptone Water (Oxoid, Basingstoke, Hampshire, UK) for pre-enrichment. Additionally, we collected 22 water samples, one from each of the 22 water bodies surveyed (Fig 1). For each sample, 5 mL of surface water, as bats are reported to come into contact with the surface of the water bodies during drinking, was drawn from five different points within the same water body using the boat, maintaining the proper biosafety and biosecurity to avoid contamination and combined to form a pooled 25 mL sample, which was then transferred into a Falcon tube containing 25 mL of Buffered Peptone Water. All samples were stored in an ice box and transported to the laboratory for analysis within 24 hours. The data collection questionnaire is provided in the supporting information (S1 File).

### Laboratory investigation

#### Isolation and identification of *Salmonella* spp.

Following enrichment, 0.1 mL of each sample was inoculated onto xylose lysine deoxycholate (XLD; Oxoid, UK) and brilliant green agar (BGA) agar plate and incubated overnight at 37°C [48,49]. Characteristic black-centered colonies with reddish zones on the XLD and red-colored colonies on BGA were selected, as the presence of *Salmonella* spp. [50]. Isolates were subcultured on nutrient agar medium and performed biochemical testing on triple sugar iron (TSI) agar slant [51] and carbohydrate fermentation test [52] to confirm the presence of *Salmonella* spp. Culture-positive and biochemical test-positive *Salmonella* spp. isolates were further confirmed by PCR using *Salmonella* genus-specific primers, ST-11 (5′-AGCCAACCATTGCTAAATTGGCGCA-3′) and ST-15 (5′-TGGTAGAAATTCCCAGCGGGTACTG-3′) [50,53]. Isolation outcomes, including positive and negative results, from both fecal and water samples, were recorded and are provided in the supplementary dataset (S1 Data)

#### Antimicrobial Susceptibility Testing (AST).

The antimicrobial resistance (AMR) profiles of all isolated *Salmonella* spp. were determined using the Kirby-Bauer disk diffusion method on Mueller-Hinton agar, in accordance with the Clinical and Laboratory Standards Institute’s (CLSI) M100 guideline, 28th edition [54]. A total of twelve antimicrobials from nine different groups named β-lactam penicillin: amoxicillin-clavulanic acid (AMC-10 µg), macrolides: azithromycin (AZM-15 µg), phenicol: chloramphenicol (C-30 µg), quinolone: ciprofloxacin (CIP-5 µg), NA = nalidixic Acid(NA-30 µg), aminoglycoside: gentamicin (CN-10 µg), cephalosporins: cefixime (CFM-5 µg), ceftriaxone (CRO-30 µg), cefotaxime (CTX-30 µg), carbapenem: imipenem (IMP-10 µg), folate antagonist: sulfamethoxazole-trimethoprim (SXT-25 µg), tetracycline: tetracycline (TE-30 µg) were used to detect AMR pattern of *Salmonella* spp*.* Inhibition zone diameters were measured and interpreted as resistant, intermediate, or susceptible in accordance with the CLSI M100 guideline, 28th edition [54]. AMR *Salmonella* spp. isolates were identified as MDR when three or more antibiotic classes showed resistant patterns, following the definition of MDR described by Magiorakos, Srinivasan [55].

### Statistical analysis

Data were checked for integrity, cleaned, and coded in MS Excel 2013, and analyzed in R software (R version 4.2.0) [56]. The prevalence of microorganisms was calculated, and the results were expressed as percentages with a 95% confidence interval (CI). The data were stratified according to the sampling sites, habitat (urban vs rural), species (*P. medius vs R. leschenaultii*), and season (winter vs summer). A Fisher’s exact 2-tailed test was done, and the level of significance was set at p < 0.05 to find the association between explanatory variables and the prevalence of bacteria. Variables identified as significant in univariable analysis were subsequently entered into a multivariable logistic regression model. The model was checked for multicollinearity and interaction following standard statistical procedures [57]. Results were expressed as odds ratios (OR) with 95% CIs. A bootstrap balanced resampling sensitivity analysis was conducted to evaluate the robustness of the estimated OR from the logistic regression. The balanced resampling analysis randomly down-samples the larger group to the size of the smaller group from a variable, and the multivariable logistic regression model was refitted [58]. The resulting ORs were compared with those from the original analysis to assess the stability of the regression estimates.

## Results

### Isolation prevalence of *Salmonella* spp. in fecal samples of fruit bats

In our study, we identified 29 positive *Salmonella* spp. isolates out of 369 fecal samples in bacterial culture. All positive *Salmonella* spp. isolates were further found to be positive by PCR. The overall isolation prevalence of *Salmonella* spp. was 7.9% (95% CI: 5.3–11.1) in the fecal samples of bat species.

### Prevalence of antimicrobial-resistant *Salmonella* spp. in fecal samples of fruit bats

In our study, we identified that 29 fecal samples were positive for *Salmonella* spp., corresponding to an overall prevalence of 7.9% (95% CI: 5.3–11.1). All *Salmonella* spp. isolates recovered by bacterial culture and confirmed by PCR exhibited resistance to at least one antimicrobial agent, resulting in a proportion of antimicrobial-resistant isolates of 100% (29/29). Therefore, the overall prevalence of antimicrobial-resistant *Salmonella* spp. at the sample level was also 7.9% (29/365; 95% CI: 5.3–11.1). Among species, *P. medius* showed a higher presence of isolates (10.9%, 95% CI: 7.2–15.7) compared to *R. leschenaultii* (2.9%, 95% CI: 0.8–7.2). The presence of resistant *Salmonella* spp. isolates was identified as higher in the summer (31.4%, 95% CI: 16.9–49.3) than in the winter (5.4%, 95% CI: 3.2–8.4). Among the locations of bat roosts, the detection of resistant *Salmonella* spp. was higher in Dhaka (12.3%, 95% CI: 7.9–18.1) followed by Madaripur (6%, 95% CI: 1.3–16.5), Faridpur (3.3%, 95% CI: 0.7–9.4), and Rajbari (2%, 95% CI: 0.1–10.6). In our study, we found a higher presence of resistant *Salmonella* spp. in urban areas (12.3%, 95% CI: 7.9–18.1) than in rural areas (3.7%, 95% CI: 1.5–7.4) (Fig 2 and Table 1).

**Table 1:** Univariable association of the presence of AMR *Salmonella* spp. in the fecal samples of bat species with associated demographic factors.

Variable	Category (N)	Presence of AMR *Salmonella* spp.			
		Positive, n (%)	95% CI	Fisher’s Exact (p-value)	OR (95% CI)
Season	Summer (35)	11 (31.4)	16.9-49.3	<0.01	8.1 (3.41-18.96)
	Winter (334)	18 (5.4)	3.2-8.4		Ref
Location^†^	Dhaka (179)	22 (12.3)	7.9-18.1	0.02	6.9 (0.9-52.25)
	Faridpur (90)	3 (3.3)	0.7-9.4		1.7 (0.17-16.69)
	Madaripur (50)	3 (6.0)	1.3-16.5		3.1 (0.31-31.14)
	Rajbari (50)	1 (2.0)	0.1-10.6		Ref
Land type	Urban (179)	22 (12.3)	7.9-18.1	<0.01	3.7 (1.52-8.8)
	Rural (190)	7 (3.7)	1.5-7.4		Ref
Species	*P. medius* (229)	25 (10.9)	7.2-15.7	<0.01	4.2 (1.42-12.24)
	*R. leschenaultii* (140)	4 (2.9)	0.8-7.2		Ref

AMR = Antimicrobial Resistance % = Percentages, CI = Confidence Interval, OR = Odds Ratio, Ref = Reference.

† Post hoc pairwise tests with Bonferroni correction for multiple comparisons showed no statistically significant pairwise differences between locations (all *p* > 0.05). Adjusted *p*-values were: Dhaka-Faridpur = 0.240, Dhaka-Madaripur = 1.000, Dhaka-Rajbari = 0.438, Faridpur-Madaripur = 1.000, Faridpur-Rajbari = 1.000, and Madaripur-Rajbari = 1.000.

**Fig 2:**
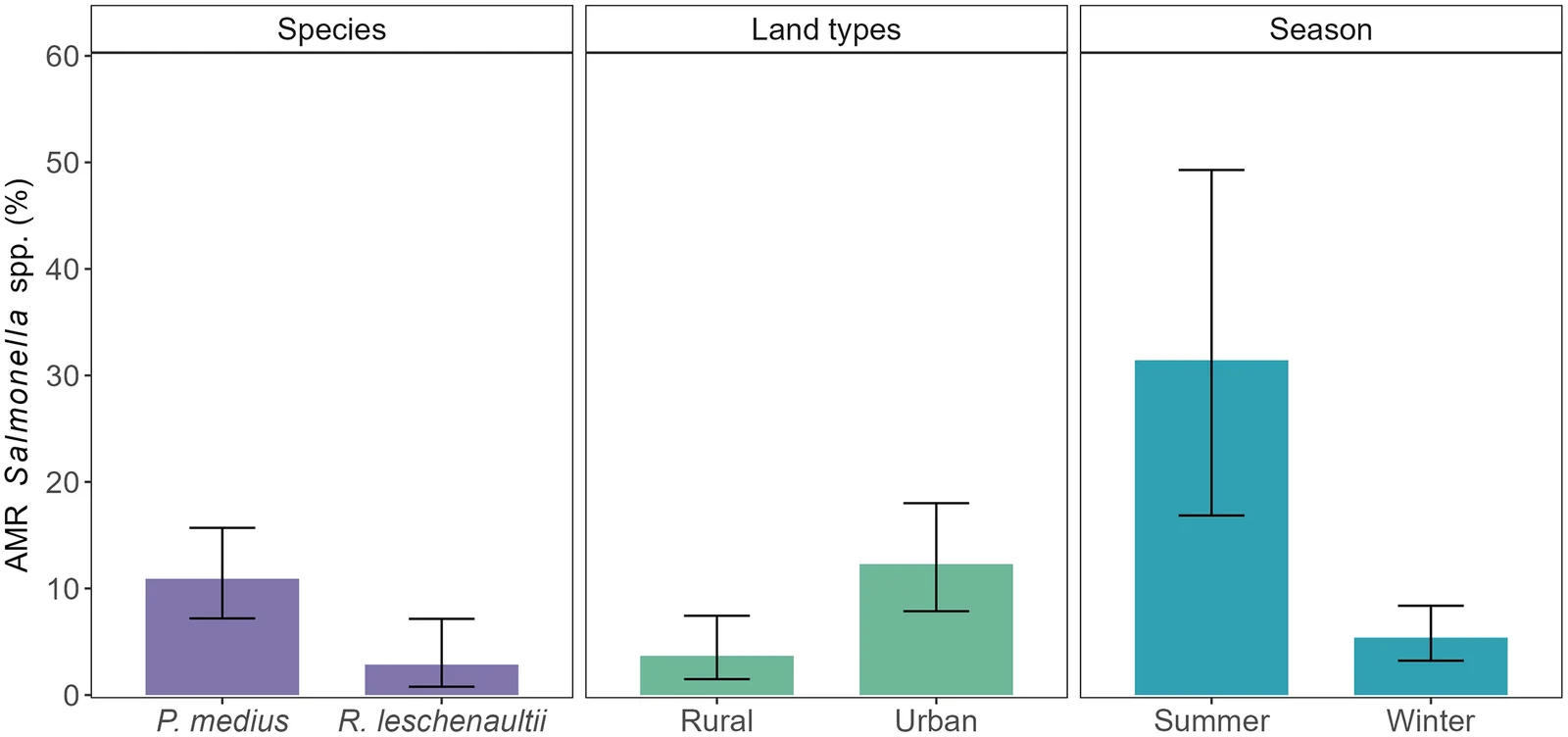
Percentages of AMR *Salmonella* spp. in bat species, land types, and seasons.

### Prevalence of antimicrobial-resistant *Salmonella* spp. in water samples by season and landscape

Antimicrobial-resistant *Salmonella* spp. isolates were detected in 31.8% (7/22; 95% CI: 13.9–54.9) of environmental water samples. When stratified by season, the prevalence of AMR *in Salmonella* spp. was 75% (95% CI: 19.4–99.4) in winter and 22.2% (95% CI: 6.4–47.6) in summer. Among the water samples, 75% (95% CI: 19.4–99.4) collected from rural areas were positive for antimicrobial-resistant *Salmonella* spp., whereas 22.2% (95% CI: 6.4–47.6) of urban water samples tested positive for resistant *Salmonella* spp. isolates (Fig 3).

**Fig 3:**
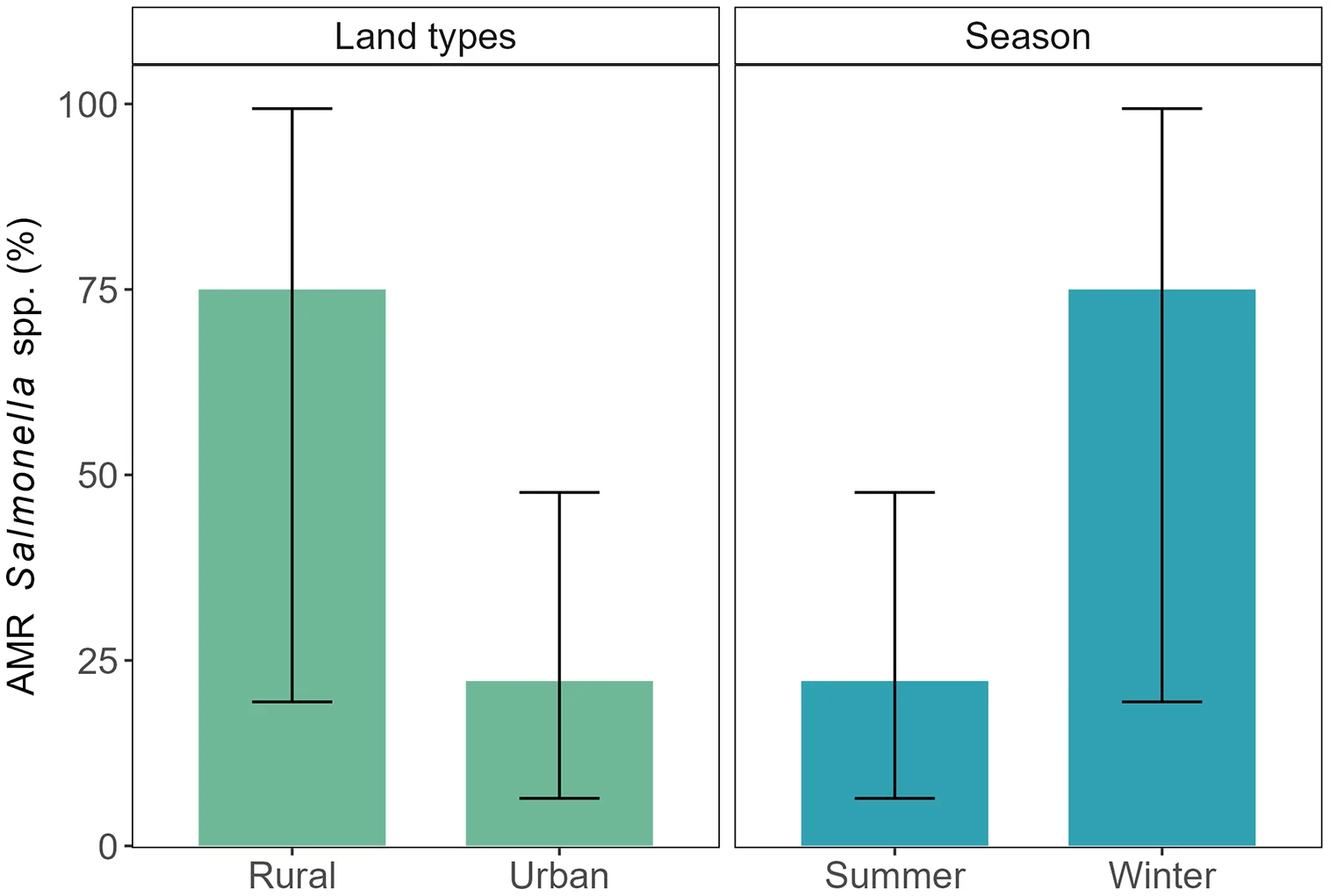
Percentages of resistant *Salmonella* spp. in water samples.

### AMR pattern of identified *Salmonella* spp

#### AMR patterns of *Salmonella* spp. in bat samples.

All identified *Salmonella* spp. in bacterial culture, and PCR showed the AMR pattern against at least one antimicrobial agent in our study. Among the antimicrobial agents, *Salmonella* spp. isolates were identified as the most resistant against tetracycline (93%), followed by nalidixic acid (86%), sulfamethoxazole-trimethoprim (80%), and azithromycin (76%). Besides, *Salmonella* spp. isolates exhibited the highest sensitivity against gentamycin (72%), followed by imipenem (69%), ceftriaxone (66%), and cefixime (62%) (Fig 4). In our study, we didn’t find any intermediate susceptibility to tetracycline, sulphamethoxazole-trimethoprim, and azithromycin. The intermediate resistance levels for the other antibiotics ranged from 3% to 27%, except for Ciprofloxacin (76%). However, we repeated the evaluation of *Salmonella* spp*.* susceptibility. isolates to ciprofloxacin, and we obtained the same results.

**Fig 4:**
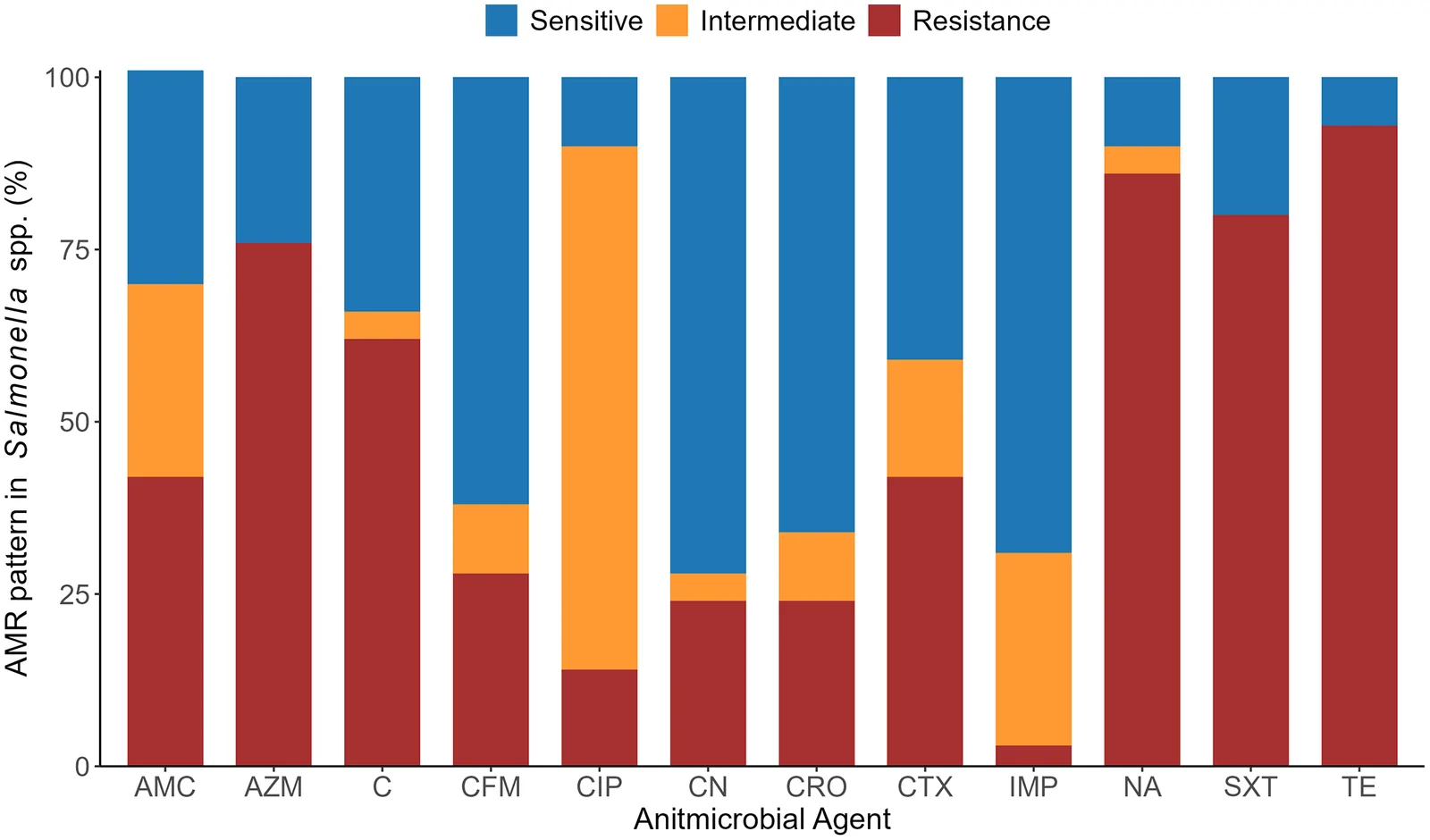
Antimicrobial resistance patterns of *Salmonella* spp. in fecal samples of bats. Here, AMC = Amoxicillin-Clavulanic acid-10 µg, AZM = Azithromycin-15 µg, C = Chloramphenicol-30 µg, CFM = Cefixime-5 µg, CIP = Ciprofloxacin-5 µg), CN = Gentamicin-10 µg, CRO = Ceftriaxone-30 µg, CTX = Cefotaxime-30 µg, IMP = Imipenem-10 µg, NA = Nalidixic Acid-30 µg, SXT = Sulfamethoxazole-trimethoprim-25 µg, TE = Tetracycline-30 µg.

#### AMR patterns of *Salmonella* spp. in water samples.

All identified *Salmonella* spp. isolates in the water samples showed the AMR pattern against at least one antimicrobial agent in the present study. Among the antimicrobial agents, *Salmonella* spp. isolates were identified as the most resistant against tetracycline (86%), followed by amoxicillin-clavulanic acid (75%), azithromycin (73%), ceftriaxone (72%), and ciprofloxacin (71%). On the other hand, *Salmonella* spp. isolates exhibited the highest sensitivity against both imipenem (55%), and cefotaxime (55%), followed by nalidixic acid and cefixime (52%) (Fig 5). The overall intermediate resistance to the antibiotics used ranged from 4% to 18%.

**Fig 5:**
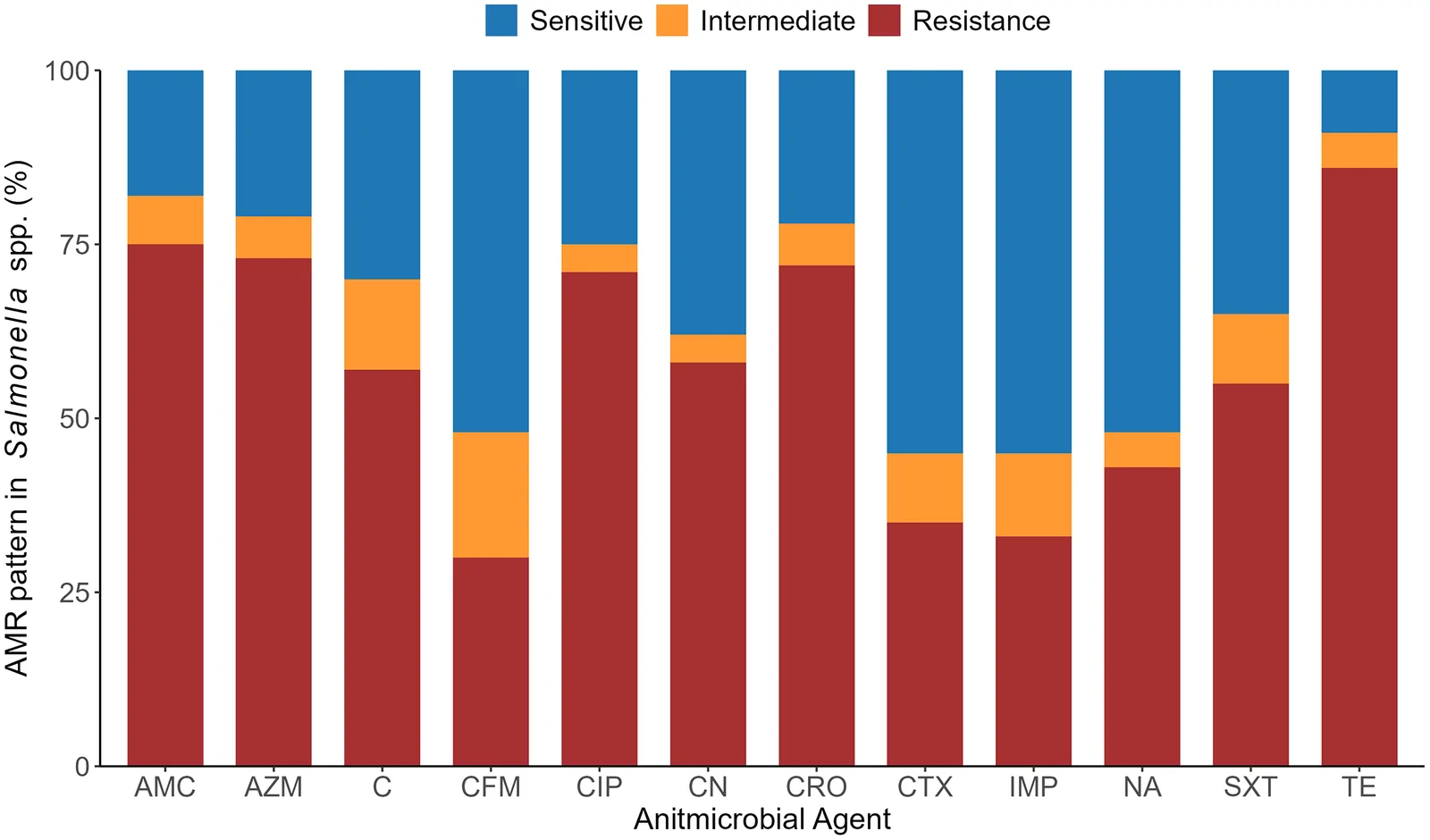
Antimicrobial resistant pattern of *Salmonella* spp. in water samples with bat interaction. Here, AMC = Amoxicillin-Clavulanic acid-10 µg, AZM = Azithromycin-15 µg, C = Chloramphenicol-30 µg, CFM = Cefixime-5 µg, CIP = Ciprofloxacin-5 µg), CN = Gentamicin-10 µg, CRO = Ceftriaxone-30 µg, CTX = Cefotaxime-30 µg, IMP = Imipenem-10 µg, NA = Nalidixic Acid-30 µg, SXT = Sulfamethoxazole-trimethoprim-25 µg, TE = Tetracycline-30 µg.

### Multidrug-resistant (MDR) patterns of *Salmonella* spp. in bat samples

In our study, 96.5% of AMR *Salmonella* spp. isolates were shown to have MDR phenotypic characteristics in our study (Fig 6). Among the identified MDR *Salmonella* spp. isolates, the higher number of isolates [9] exhibited MDR against six antimicrobial agents, followed by MDR against five antimicrobial agents (7 isolates) and three antimicrobial agents (5 isolates) (Table 2).

**Table 2:** Multidrug-resistant (MDR) patterns of *Salmonella* spp. in bat samples.

Number of antimicrobial classes	Pattern of Resistance	Frequency
Three class	TE-AMC-AZM	2
	TE-NA-SXT	1
	TE-CRO/CFM/CTX-SXT	1
	TE-NA/CIP-AZM	1
Four class	C-CTX-AMC-AZM	1
	TE-NA-SXT-C	1
	TE-NA-SXT-CFM	1
Five class	TE-NA-SXT-AMC-AZM	1
	TE-NA-SXT-C-CTX	1
	TE-NA/CIP-CRO/CFM/CTX-SXT-C	1
	TE-NA/CIP-SXT-C-AZM	3
	TE-NA-SXT-AMC-AZM	1
Six class	TE-NA-SXT-CTX-AMC-AZM	1
	TE-NA-CN-IMP-C-AZM	1
	TE-NA-SXT-C-AMC-AZM	1
	TE-NA-C-CTX-AMC-AZM	1
	TE-NA-SXT-CN-C-AZM	2
	TE-NA/CIP-SXT-CFM/CTX/CFM-C-AZM	2
	NA-SXT-CN-CTX-AMC-AZM	1
Seven class	TE-NA-SXT-CN-C-AMC-AZM	1
	TE-NA-CRO/CFM/CTX-SXT-C-AZM-AZM	1
	TE-NA-CRO/CFM/CTX-SXT-CN-C-AZM	1
Eight class	TE-NA/CIP-CRO/CFM/CTX-SXT-CN-C-AMC-AZM	1

**Fig 6:**
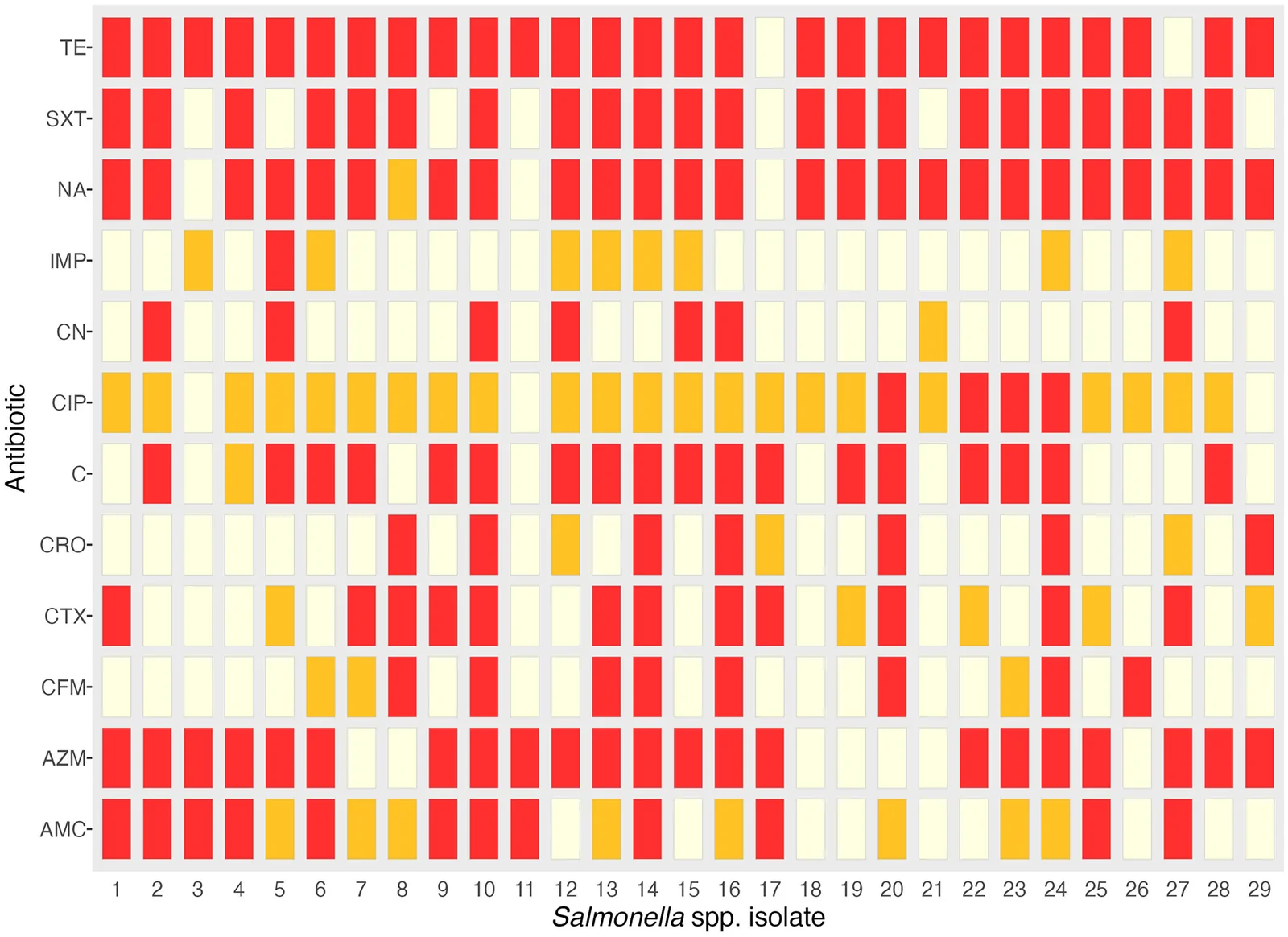
MDR pattern of isolated *Salmonella* spp. from bats. Here, AMC = Amoxicillin-Clavulanic acid-10 µg, AZM = Azithromycin-15 µg, C = Chloramphenicol-30 µg, CFM = Cefixime-5 µg, CIP = Ciprofloxacin-5 µg), CN = Gentamicin-10 µg, CRO = Ceftriaxone-30 µg, CTX = Cefotaxime-30 µg, IMP = Imipenem-10 µg, NA = Nalidixic Acid-30 µg, SXT = Sulfamethoxazole-trimethoprim-25 µg, TE = Tetracycline-30 µg; Red color represents resistant, Yellow represents intermediate, and White represents sensitive.

### Risk factors associated with MDR *Salmonella* spp. in bat samples

The dataset included four explanatory variables considered as potential risk factors for MDR *Salmonella* spp. Cramer’s V analysis indicated a high level of association with the variables District and Land type (S1 Table). These variables were excluded from the final logistic regression analysis to avoid multicollinearity. The interaction term between Season and Species did not show a significant effect and was not retained in the model (S2 Table). Logistic regression (LR) analysis identified the season of sample collection as a potential risk factor, with the odds of detecting MDR *Salmonella* spp. being 5.1 times higher during summer than in winter. No significant association was observed between the presence of MDR *Salmonella* spp. and bat species (Table 3). The stability of the estimated odds ratio was evaluated using bootstrap balanced resampling sensitivity analysis (1000 iterations) since the number of samples in the winter season were highly skewed. The estimated effect of season remained consistent in the analysis (S3 Table).

**Table 3:** Multivariable logistic regression model in the presence of MDR *Salmonella* spp. in bat samples in Bangladesh.

Variable	Category	Coefficient estimate	OR (95% CI)	P value
	Intercept	−2.6		<0.01
Season	Winter	Ref	Ref	
	Summer	1.6	5.1 (2.0–12.8)	<0.01
Species	*P. medius*	Ref	Ref	
	*R. leschenaultii*	−0.97	0.4 (0.1–1.1)	0.09

Ref = Reference, OR = Odds Ratio, CI = Confidence Interval.

## Discussion

This study investigated the prevalence and patterns of AMR in *Salmonella* spp. isolated from bat fecal samples and their surrounding water habitats within the human-livestock-wildlife interface in Bangladesh. Importantly, we identified MDR *Salmonella* spp. from the bat samples, highlighting a potential public health concern. Our findings indicate that the presence of AMR *Salmonella* spp. in bats is influenced by potential ecological factors such as season.

The overall presence of AMR *Salmonella* spp. in bat samples in this study was low, aligning with previous limited studies in Bangladesh. For instance, Islam [59] the presence of AMR *Salmonella* spp. in fruit bats (*R. leschenaultii*) was reported at around 8%, which is similar to our findings. However, an earlier study by Islam et al. [37] reported no presence of AMR *Salmonella* spp. in fruit bats, and one positive isolate of *Salmonella* spp. was identified from the rectal swabs of *P. giganteus* in Bangladesh. Globally, research on AMR *Salmonella* in bats remains scarce, with most studies focusing on virology and parasitology [60]. The presence of *Salmonella* spp. has been reported in fruit bats in different countries [61]. Also, *Salmonella* spp. has been identified in other bat species, including the insectivorous and fish-eating bats, with low prevalence [25,26,62].

Despite limited evidence of AMR *Salmonella* spp. in bats, several other wild animals, including squirrels [63], rhesus macaques, and wild birds, have previously been documented to harbor AMR *Salmonella* spp. in Bangladesh [50,64]. Fruit bats often roost near human settlements, livestock farms, and other wild animals, sharing the same food and water resources. This potential ecological overlap may be associated with the co-occurrence of AMR bacteria across the One Health interface [65]. In Bangladesh, outbreaks of *Salmonella* Typhi in humans have been linked to contaminated fruits such as papayas, which may be exposed to bat and bird feces [36]. Besides, the microbial burden among the different bat populations may be influenced by long-distance migrations and frequent intra- and inter-roost movements of bat species within the colonies [66,67].

The current study has identified the presence of AMR *Salmonella* spp. in water samples collected from water bodies such as ponds, lakes, and swamps near bat roosts, where bats frequently visited to drink water. Both humans and livestock commonly use these stagnant water bodies in rural Bangladesh and are often contaminated with sewage, animal waste, or runoff from poultry farms [38,68]. Such contamination likely introduces *Salmonella* spp. from typhoid patients or from poultry treated with antimicrobials [69]. Bats can contract resistant *Salmonella* spp. from the same water source by drinking contaminated water, as they are observed sharing the same water bodies. Antimicrobials and AMR bacteria can be excreted in active form into the environment and sewage through defecation and urination from treated animals and humans [70], where resistant bacteria can also proliferate and may pose a risk of AMR bacterial co-occurrence to other wild animals, including bats. A higher proportion of antimicrobial-resistant *Salmonella* spp. was observed in rural water bodies compared with urban areas, although the difference was not statistically significant. The observed pattern may still reflect differences in human and animal interactions with water sources, with rural water bodies more frequently used for bathing and for washing utensils, as well as by animals. In contrast, people in urban areas tend to be more cautious and generally avoid using natural water bodies for such purposes.

In this study, we observed that *Salmonella* spp. isolates of both bat samples and water samples showed resistance against multiple antimicrobial agents. However, previous studies on fruit bats in Bangladesh [37] and Australia [61] have identified *Salmonella* spp. isolates that show no resistance to any antibiotics. The variation in the results may be due to spatiotemporal contrast across the different studies. However, Adesiyun et al. [26] found that *Salmonella* spp. isolates are highly resistant to antimicrobial agents such as macrolides, similar to our finding that azithromycin showed resistance in *Salmonella* spp. A high percentage of tetracycline-resistant *Salmonella* spp. has been identified in the poultry industry in Bangladesh [68]. This might explain the higher percentages of tetracycline-resistant *Salmonella* spp. detected in bats, which are transmitted from tetracycline-treated animals to bats through direct or indirect contact with animals’ waste products. Besides, tetracycline-resistant *Salmonella* spp. have been identified in different water bodies, including sewage, rivers, and ponds in Bangladesh, supporting the findings of our present study [71]. Waste products and litter of poultry contain tetracycline-resistant *Salmonella* spp. [72], which may also contaminate the common water habitat of bats, livestock, and humans by being thrown into the water bodies, and acts as a potential source of AMR-resistant *Salmonella* spp. co-occurrence in bats by drinking the contaminated water. In our study, ciprofloxacin- and amoxicillin-resistant *Salmonella* spp. have been identified in water samples near the bat’s roost, which may be associated with the potential ecological overlap of AMR-resistant *Salmonella* spp. from other contaminated sources. Resistant strains of *Salmonella* Typhi to ciprofloxacin and amoxicillin have been reported in typhoid patients in Bangladesh [73], and resistant bacteria excreted by patients, subsequently dispersed in the environment, such as sewage systems due to inadequate wastewater management in hospital settings in Bangladesh [74], and contaminated the water bodies with fecal materials through sewage [12]. Our analysis identified season as a significant ecological factor influencing the potential to detect AMR in *Salmonella* spp. isolates in bats. Some bat species enter hibernation or torpor in winter, resulting in reduced body temperature and metabolic rate due to food scarcity [75]. This reduces their mobility and the likelihood of pathogen transmission. In contrast, bats remain more active and show significantly higher thermoregulatory, locomotion, and communicative behavior in summer than in the winter season [76]. Bat aggregation within a roost is significant because it increases the risk of bacterial transmission through direct contact among individuals.

This study underscores the role of bats and their shared water environments in the ecology of AMR *Salmonella* spp. within the One Health context in Bangladesh. Although the overall prevalence was low, the detection of MDR *Salmonella* spp. in both bat and water samples highlights the potential ecological overlap for environmental and interspecies transmission of resistant pathogens. These findings call for integrated surveillance systems that monitor AMR at the human–animal–environment interface and promote responsible antimicrobial use in both healthcare and agricultural settings to reduce the risk of co-occurrence of resistant microorganisms across ecological boundaries. While this study provides insight into the ecology and epidemiology of *Salmonella* spp. in bats in Bangladesh, several limitations have been reported for further exploration. The sample size was calculated using a conservative 50% expected prevalence due to limited data on AMR *Salmonella* spp. in Bangladesh and neighboring countries. Sampling relied on convenience and judgment-based approaches because the two bat species occupied separate habitats and logistical constraints limited field sampling, which may limit species-specific inferences. In addition, substantially fewer fecal samples were collected during the summer and monsoon seasons than during winter due to field sampling constraints, potentially reducing the precision of seasonal comparisons. To evaluate the potential impact of this imbalance, we conducted a bootstrap-based sensitivity analysis, which showed that the principal regression estimates remained stable despite unequal seasonal sampling effort.

Fecal samples were collected non-invasively beneath bat roosts; individual bats were not captured or genetically identified. Thus, the samples should be considered environmental fecal deposits rather than confirmed samples from individual bats, and sample independence cannot be fully guaranteed. Consequently, the prevalence estimates should be interpreted with caution, as they reflect the proportion of fecal samples testing positive and should not be regarded as precise estimates of individual-level infection prevalence. The observed associations should likewise be interpreted with caution, as potential clustering of samples by individual bats or roosts may compromise the independence of observations. Furthermore, the cross-sectional study design does not permit inference of transmission pathways or causal relationships between bats and the co-occurrence of multidrug-resistant *Salmonella* spp. Molecular characterization of antimicrobial resistance genes and serovar identification of the recovered of *Salmonella* spp. isolates were beyond the scope of this study. However, these analyses could have provided greater epidemiological resolution and strengthened interpretation of the diversity and relatedness of the isolates. Future investigations incorporating whole-genome sequencing, antimicrobial resistance gene profiling, and serotyping could provide greater insight into the genetic diversity, resistance mechanisms, and epidemiological relationships of *Salmonella* spp. circulating at the human–livestock–wildlife interface, thereby strengthening One Health surveillance and informing evidence-based control strategies.

## Conclusion

This study demonstrated the presence of MDR *Salmonella* spp. in both fruit bats and associated environmental water sources near bat roosts in Bangladesh, highlighting the ecological and epidemiological baseline of AMR at the human-environment-wildlife interface. The detection of MDR *Salmonella* spp. across both fruit bats and water samples underscores the epidemiological relevance of wildlife as a component of the broader AMR landscape in South Asia, where surveillance data from non-clinical and non-agricultural reservoirs remain critically scarce. The extensive and frequently indiscriminate application of antimicrobials in humans and animal populations, combined with environmental pollution, probably has an association in the emergence and proliferation of AMR bacteria and MDR development within the bat population. Insufficient wastewater management at hospitals and animal farms can increase bats’ exposure to resistant bacteria through contaminated drinking water from shared water sources. The possible roles of bats as co-occurrences and carriers of AMR highlight the importance of focused ecological monitoring and a more comprehensive understanding of the factors driving antimicrobial resistance at the wildlife-environment interface. Additional studies are suggested to investigate MDR bacteria and resistance genes associated with bats, and to examine disease transmission pathways to understand the impact of AMR bacteria in wildlife across Bangladesh. Simultaneously, enhanced public health awareness and responsible use of antimicrobials among human populations and livestock producers should be accelerated to mitigate the development of MDR, which can lead to catastrophic consequences for human and animal health.

Overall, this study reinforces that antimicrobial resistance extends beyond clinical settings and should be considered a One Health challenge involving interconnected human, animal, wildlife, and environmental systems. Coordinated One Health surveillance and evidence-based interventions will be essential to preserve antimicrobial effectiveness and protect public, animal, and ecosystem health in Bangladesh and other regions facing similar ecological and public health challenges.

## Supporting information

S1 File The questionnaire used for collecting ecological information. (DOCX)

S2 File PLOS questionnaire on inclusivity in global research. (DOCX)

S1 Table Cramer’s V output between the variables used in multivariable logistic regression. (DOCX)

S2 Table Likelihood ratio test comparing logistic regression models with and without the interaction terms. (DOCX)

S3 Table Comparison of odds ratios from the original logistic regression model and the bootstrap balanced resampling sensitivity analysis (1,000 iterations and seed = 123). (DOCX)

S1 Data The sampled dataset. (XLSX)
