# Supplementary material for: Ecology and epidemiology of multidrug resistance Salmonella in fruit Bats and shared water sources in Bangladesh
Source: PLoS One. 2026 Oct 30;21(10):e0361701. doi: 10.1371/journal.pone.0361701 (PMC13722153; doi:10.1371/journal.pone.0361701)
Supplement: Supplementary file 1 [file pone.0361701.s001.docx]

**Observational Questionnaire:** Prevalence and risk factors of antimicrobial resistance patterns of *Salmonella* spp. in fruit bats and shared water sources at human-animal interfaces in Bangladesh.

**Section A: General information**

1. Date of Interview: ___ / ___ / ___
2. Name of the sampling site : ----------------------
3. GPS Coordinates: Latitude: ____________ Longitude: ____________

**Section B: Ecological and environmental information (Ecological observation)**

1. What types of buildings or infrastructure are present near the capturing site?

Hospitals or clinics/ Animal farms or livestock sheds/ markets/ Residential homes/ Jungle

2. What is the level of human activity in the capturing area?

Low / Moderate / High

3. Are there any water bodies surrounding the bat roost?

A) Yes	B) No

4. If yes, what type of water bodies?
A) Pond B) River C) Lake D) Wetland E) Lagoon
5. Is the water body used by humans?

A) Yes	B) No

6. If yes, what type of activities are observed during the sampling?

A) Bathing B) Playing C) Swimming D) Collecting drinking water E) Fishing E) Washing clothes F) Others

7. Is the water body used by domestic animals?

A) Yes	B) No

8. If yes, what type of activities are observed during the sampling?

A) Bathing B) Swimming C) Drinking water D) Others

9. Is the water body used by wild animals?

A) Yes	B) No

10. If yes, what type of activities are observed during the sampling?

A) Bathing B) Swimming C) Drinking water D) Others

**Section C: Bat Capture Data (Researcher only)**

1. Species Captured:
2. Sex: Male / Female
3. Age Group: Juvenile / Adult
4. Body Condition Score (BCS): Good / Fair/ Poor
5. Sample Collected: Oral swab / Rectal swab / Urine swab

**Section D: Morphometric and demographic characteristics (Researcher only)**

1. Body weight (g): _______________
2. Body length (cm): _______________
3. Forehead length (cm): _______________
4. Ear length (cm): _______________
5. Health condition: Apparently healthy/Sick/ Diseased
6. Visible wounds or lesions on the body?
