# Supplementary material for: Ecology and epidemiology of multidrug resistance Salmonella in fruit Bats and shared water sources in Bangladesh
Source: PLoS One. 2026 Oct 30;21(10):e0361701. doi: 10.1371/journal.pone.0361701 (PMC13722153; doi:10.1371/journal.pone.0361701)
Supplement: Supplementary file 3 [file pone.0361701.s003.docx]

**Supplementary Table 1:** Cramer’s V output between the variables used in multivariable logistic regression. Value greater than 0.4 is considered collinear.

| **Variable 1** | **Variable 2** | **Cramér's V** |
| --- | --- | --- |
| District | Land type | 1 |
| District | Season | 0.33 |
| District | Species | 1 |
| Land type | Season | 0.32 |
| Land type | Species | 0.75 |
| Season | Species | 0.24 |
