# Supplementary material for: Ecology and epidemiology of multidrug resistance Salmonella in fruit Bats and shared water sources in Bangladesh
Source: PLoS One. 2026 Oct 30;21(10):e0361701. doi: 10.1371/journal.pone.0361701 (PMC13722153; doi:10.1371/journal.pone.0361701)
Supplement: Supplementary file 4 [file pone.0361701.s004.docx]

**Supplementary Table 2:** Likelihood ratio test comparing logistic regression models with and without the interaction terms (Season, Species).

| **Resid.Df** | **Resid.Dev** | **Df** | **Deviance** | **Pr(>Chi)** |
| --- | --- | --- | --- | --- |
| 366 | 178.78 |  |  |  |
| 366 | 178.78 | 0 | 0 | - |
