# Supplementary material for: Ecology and epidemiology of multidrug resistance Salmonella in fruit Bats and shared water sources in Bangladesh
Source: PLoS One. 2026 Oct 30;21(10):e0361701. doi: 10.1371/journal.pone.0361701 (PMC13722153; doi:10.1371/journal.pone.0361701)
Supplement: Supplementary file 5 [file pone.0361701.s005.docx]

**Supplementary Table 3:** Comparison of odds ratios from the original logistic regression model and the bootstrap balanced resampling sensitivity analysis (1,000 iterations and seed =123).

| **Variable** | **Original OR** | **95% CI** | **p-value** | **Bootstrapped mean OR** | **95% CI** |
| --- | --- | --- | --- | --- | --- |
| Season (winter vs summer) | 5.1 | 2.0 – 12.8 | <0.01 | 5.1 | 1.6-9.6 |

OR: Odds ratio; CI: Confidence interval
